# Surface plasma resonant effect of gold nanoparticles on the photoelectrodes of dye-sensitized solar cells

**DOI:** 10.1186/1556-276X-8-450

**Published:** 2013-10-30

**Authors:** Teen-Hang Meen, Jenn-Kai Tsai, Shi-Mian Chao, Yu-Chien Lin, Tien-Chuan Wu, Tang-Yun Chang, Liang-Wen Ji, Walter Water, Wen-Ray Chen, I-Tseng Tang, Chien-Jung Huang

**Affiliations:** 1 Department of Electronic Engineering, National Formosa University, Hu-Wei, Yunlin 632, Taiwan; 2 Department of Electrical Engineering, Hsiuping University of Science and Technology, Taichung 412, Taiwan; 3 Institute of Electro-Optical and Materials Science, National Formosa University, Yunlin 632, Taiwan; 4 Department of Greenergy, National University of Tainan, Tainan 700, Taiwan; 5 Department of Applied Physics, National University of Kaohsiung, Kaohsiung 811, Taiwan

**Keywords:** Gold nanoparticles, Dye-sensitized solar cells, Seed-mediated growth method

## Abstract

In this study, we prepared different shapes of gold nanoparticles by seed-mediated growth method and applied them on the photoelectrodes of dye-sensitized solar cells (DSSCs) to study the surface plasma resonant (SPR) effect of gold nanoparticles on the photoelectrodes of dye-sensitized solar cells. The analyses of field emission scanning electron microscopy show that the average diameter of the spherical gold nanoparticles is 45 nm, the average length and width of the short gold nanorods were 55 and 22 nm, respectively, and the average length and width of the long gold nanorods were 55 and 14 nm, respectively. The aspect ratio of the short and long gold nanorods was about 2.5 and 4, respectively. The results of ultraviolet–visible absorption spectra show that the absorption wavelength is about 540 nm for spherical gold nanoparticles, and the absorption of the gold nanorods reveals two peaks. One is about 510 to 520 nm, and the other is about 670 and 710 nm for the short and long gold nanorods, respectively. The best conversion efficiency of the dye-sensitized solar cells with spherical gold nanoparticles and short and long gold nanorods added in is 6.77%, 7.08%, and 7.29%, respectively, and is higher than that of the cells without gold nanoparticles, which is 6.21%. This result indicates that the effect of gold nanoparticles on the photoelectrodes can increase the conductivity and reduce the recombination of charges in the photoelectrodes, resulting in the increase of conversion efficiency for DSSCs. In addition, the long gold nanorods have stronger SPR effect than the spherical gold nanoparticles and short gold nanorods at long wavelength. This may be the reason for the higher conversion efficiency of DSSCs with long gold nanorods than those of the cells with spherical gold nanoparticles and short gold nanorods.

## Background

Recently, a new type of solar cell based on dye-sensitized nanocrystalline titanium dioxide has been developed by O'Regan and Grätzel [1]. The most attractive features of this technology are reduced production costs and ease of manufacture. Dye-sensitized solar cells (DSSCs) based on nanocrystalline TiO_2_ electrodes are currently attracting widespread attention as a low-cost alternative to replace conventional inorganic photo voltaic devices [2-6]. The function of DSSCs is based upon the injection of electrons of photoexcited state of the sensitizer dye into the conduction band of the semiconductor. Constant researches attempt to achieve four goals: to promote the adsorption of dye, to harvest more solar light, to smoothen the progress of transport of photoexcited electrons, and to facilitate the diffusion of an electrolyte ion. A record of the cell convertible efficiency of 11% was achieved using N3 (RuL_2_(NCS)_2_, L = 2,2′-bipyridyl-4,4′-dicarboxylic acid) dye and the electrolyte containing guanidinium thiocyanate [7]. Grätzel et al. used DSSCs sensitized by N3 dye using guanidinium thiocyanate as self-assembly-facilitating agent, leading to improvement in efficiency [8-11]. Some of the cheaper dyes have also been used as sensitizers to improve the absorption in the visible region [12-14]. Gold nanoparticles cannot only increase the conductivity, the different shapes will result to different intensities of the surface plasma resonance (SPR) [15]. Recent studies have shown that metal or metal ion-doped semiconductor composites exhibit shift in the Fermi level to more negative potentials. Such a shift in the Fermi level improves the energetics of the composite system and enhances the efficiency of interfacial charge-transfer process [16]. In addition, Chou et al. prepared TiO_2_/nanometal composite particles by dry particle coating technique. This study shows that the power conversion efficiency *η* of the DSSCs with a film of TiO_2_/Au (or TiO_2_/Ag) on the working electrode always exceeds that of the conventional DSSCs due to the presence of the Schottky barrier [17]. In this study, we prepared different shapes of gold nanoparticles by seed-mediated growth method to apply on the photoelectrodes of the DSSCs. The gold nanoparticles and DSSCs were investigated by field emission scanning electron microscopy (FE-SEM), ultraviolet–visible (UV–vis) absorption spectra, current–voltage characteristics, electrochemical impedance spectroscopy (EIS), and incident photon conversion efficiency (IPCE) analyses to study the SPR effect of the gold nanoparticles on the photoelectrodes of the dye-sensitized solar cells.

## Methods

### Chemicals

Hydrogen tetrachloroaurate(III) trihydrate (HAuCl_4_‧3H_2_O, 99.9%), hexadecyltrimethylammonium bromide (CTAB), silver nitrate (AgNO_3_, 99.8%), ascorbic acid (AA, 99.7%), sodium borohydride (NaBH_4_, 99.9%) were used as reactants. TiO_2_ powder and 4-tert-butylpyridine were used as preparation paste of the photoelectrodes. The deionized (DI) water that was used throughout the experiments was purified using a Milli-Q system (Millipore Co., Billerica, MA, USA). Glassware was cleaned by soaking it in aqua regia and then washing it with DI water.

### Synthesis of gold nanoparticles

We used seed-mediated growth method to prepare the gold nanoparticles. This method involves two main steps: (1) preparation of seed solution, where the gold seed solution was prepared by first combining (5 mL, 0.5 mM) and CTAB (5 mL, 0.2 M), followed by the addition of freshly made NaBH_4_ (0.6 mL, 0.01 M) under vigorous stirring. Then, the mixture was left undisturbed, aged for 2 h at 25°C for further use. (2) The other is the preparation of a growth solution that consists of HAuCl_4_‧3H_2_O (5 mL, 1 mM), 0.2 mL AgNO_3_ (spherical and short and long rods are 0.01 and 0.04 M, respectively), and CTAB (5 mL, 0.2 M). AA (70 μL, 0.0788 M) was then added and followed by brief stirring (approximately 1 min). Finally, the spherical gold nanoparticles were synthesized, every 10 s, a drop for the short gold nanorods (aspect ratio of about 2.5), and every 1 min, a drop for the long gold nanorods (aspect ratio of about 4). Lastly, 25 μL of the seed solution was added to the growth solution. The mixture was allowed to react at 30°C. Centrifugation of the gold nanoparticles was carried out at 4,000 rpm for 20 min, and the supernatant was removed and then suspended with the same volume of deionized water. This process was repeated three times.

### Assembling the DSSC

We used the scraper method to prepare the photoelectrode on fluorine-doped tin oxide glass substrate. The TiO_2_ coatings were prepared from commercial TiO_2_ particles (P25). The compositions of the TiO_2_ paste were TiO_2_, 4-tert-butylpyridine, and deionized water. The concentration of the TiO_2_ paste was 10 wt.%. The concentration of the gold nanoparticles added in the TiO_2_ paste is about 1.5 wt.%. With the addition of gold nanoparticles, the TiO_2_ film was scraped to the desired thickness on the substrate by scratching. After drying, we pressed the TiO_2_ film by suitable pressure and annealed it at 450°C for 30 min to complete the photoelectrode. The size of the TiO_2_ film electrodes used was 0.25 cm^2^ (0.5 cm × 0.5 cm). Finally, we kept the photoelectrode immersed in a mixture containing a 3 × 10^-4^ M solution of N3 dye and ethyl alcohol at 45°C for 1.5 h in the oven. The electrode was assembled into a sandwich-type open cell using platinum plate as a counter electrode.

### Characterization

The surface morphology of the samples was observed using FE-SEM. The ultraviolet–visible absorption spectra of the samples were observed using a UV–vis spectrophotometer. The current–voltage characteristics and EIS of the samples were measured using Keithley 2400 source meter (Keithley Instruments Inc., Cleveland, OH, USA) and were determined under simulated sunlight with white light intensity, *P*_L_ = 100 mW/cm^2^. In the IPCE measurement, a xenon lamp (Oriel (Newport Corporation, Jiangsu, China), model 66150, 75 W) was used as the light source, and a chopper and lock-in amplifier were used for phase-sensitive detection.

## Results and discussion

**Figure 1:**
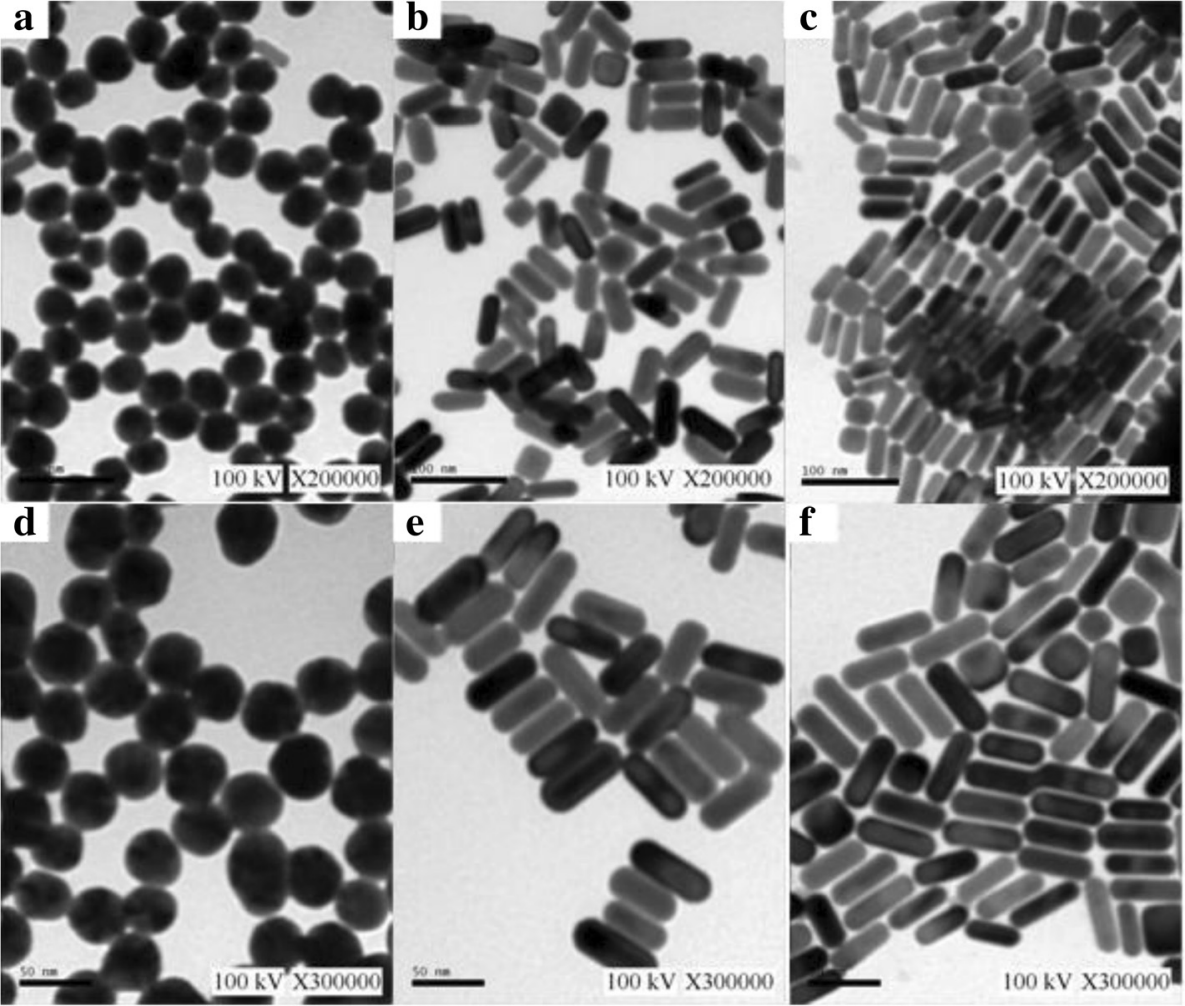
**TEM images of gold nanoparticles with different shapes. (a**, **d)** Spherical nanoparticles. **(b**, **e)** Short nanorods (aspect ratio (AR) 2.5). **(c**, **f)** Long nanorods (AR 4).

**Figure 2:**
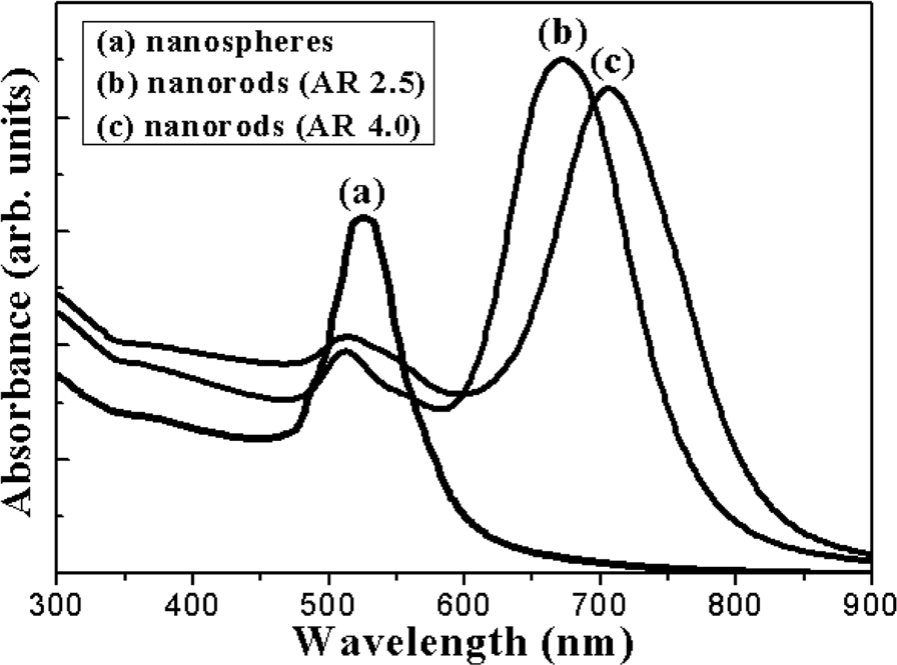
The UV–vis absorption spectra of spherical gold nanoparticles, short nanorods, and long nanorods.

**Figure 3:**
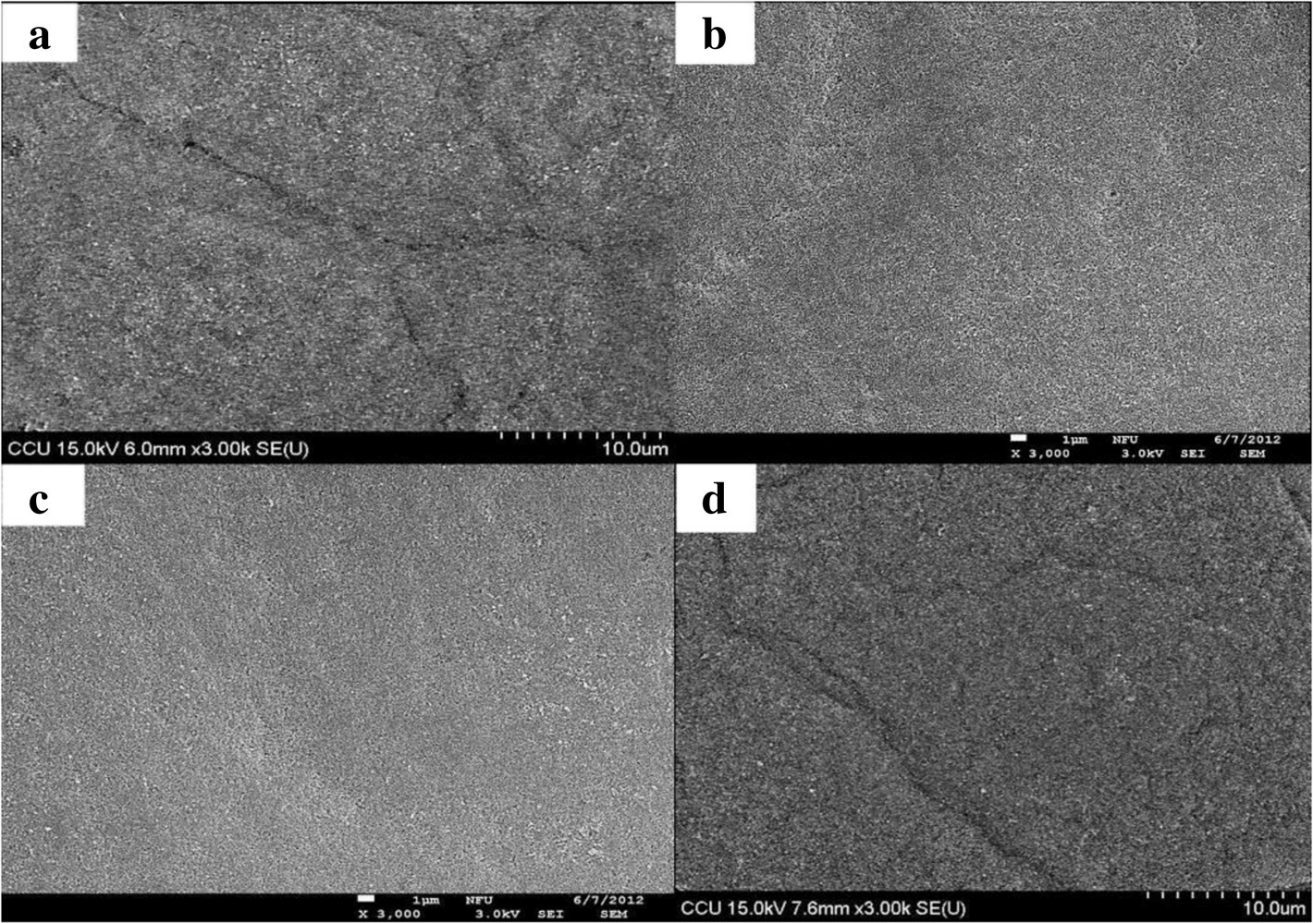
**FE-SEM images of the photoelectrodes of dye-sensitized solar cells. (a)**, **(b)**, **(c) (d)** Top view images. **(a)** Without gold nanoparticles added. **(b)** With spherical gold nanoparticles added. **(c)** With short gold nanorods added. **(d)** With long gold nanorods added.

**Figure 4:**
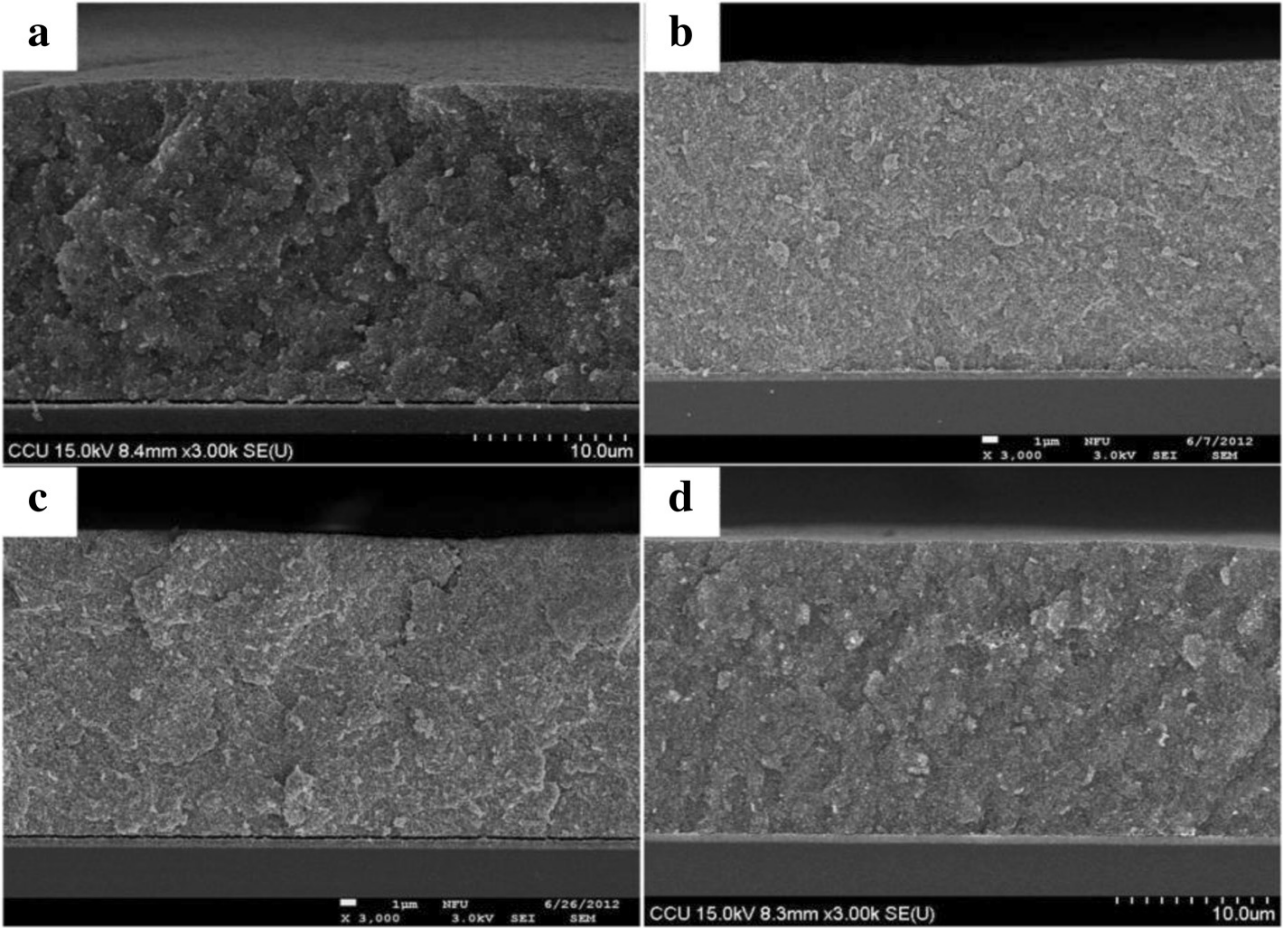
**Cross-section FE-SEM images of the photoelectrodes of dye-sensitized solar cells. (a)** Without gold nanoparticles added. **(b)** With spherical gold nanoparticles added. **(c)** With short gold nanorods added. **(d)** With long gold nanorods added.

Figure 1a,d shows the TEM images of the gold nanoparticles, which are almost spherical and uniformly dispersed with a size of about 66 nm. Figure 1b,e shows the TEM images of the short gold nanorods. It is revealed that the short gold nanorods have an aspect ratio of 2.5. Figure 1c,f shows the TEM images of the long gold nanorods. It indicates that the long gold nanorods have an aspect ratio of 4. The ultraviolet–visible absorption spectra of the gold nanoparticles are shown in Figure 2. The standard absorption wavelength is about 540 nm for the spherical gold nanoparticles. The short gold nanorods show the transverse SPR band at 510 nm and the longitudinal SPR band at 670 nm. The long gold nanorods show the transverse SPR band at 510 nm and the longitudinal SPR band at 710 nm. Figure 3 shows the FE-SEM images of the TiO_2_ films without and with gold nanoparticles added. The films are all smooth, as shown in Figures 3 and 4. Figure 4 shows the cross-section FE-SEM images of the TiO_2_ films without and with gold nanoparticles added. The thickness of these TiO_2_ films was about 22 μm.

**Figure 5:**
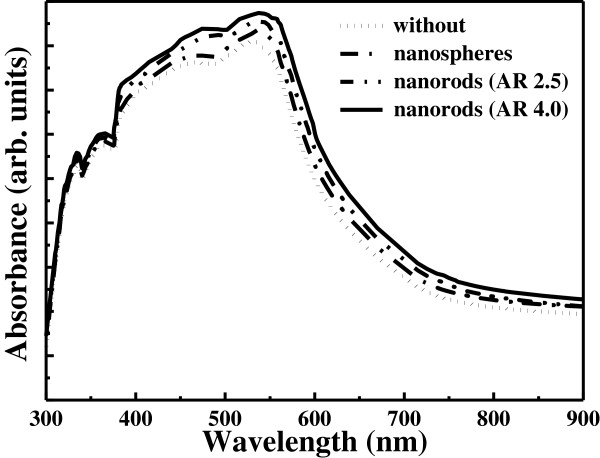
**The UV–vis absorption spectrum of TiO**_**2 **_**films without and with gold nanoparticles added.**

**Figure 6:**
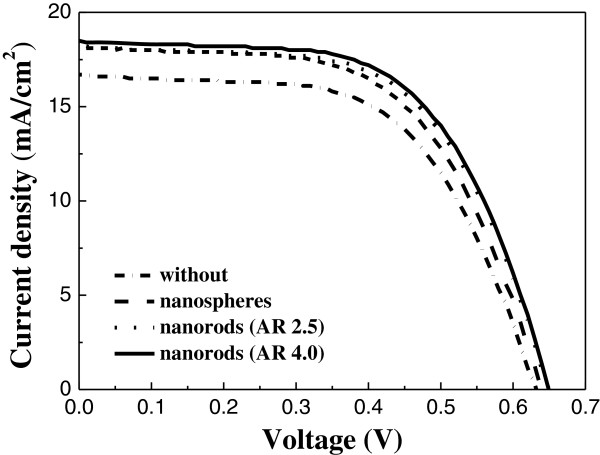
**The *J*-*V *** **curves of DSSCs without and with gold nanoparticles added.**

Figure 5 shows the UV–vis absorption spectra of the TiO_2_ films without and with gold nanoparticles added. It is found that the absorption spectrum of the TiO_2_ film with gold nanoparticles added is better than that of the film without gold nanoparticles, and the film with gold nanorods has stronger SPR intensity than that with spherical gold nanoparticles at long wavelength. Figure 6 shows the current–voltage characteristics of the DSSCs without and with nanoparticles added. The parameters for the short-circuit current density (*J*_sc_), the open circuit potential (*V*_oc_), the fill factor (F.F.), and the overall conversion efficiency (*η*) are listed in Table 1. It is noted that the *V*_oc_ of the cell with long gold nanorods is higher than those cells with spherical gold nanoparticles and short gold nanorods. This result provides an evidence to prove the reports of Subramanian et al. [16] and Chou et al. [17] and may be due to the shift in the Fermi level to more negative potentials and the presence of the Schottky barrier. From the results of Table 1, it is found that the best conversion efficiency of the dye-sensitized solar cell with long gold nanorods added is 7.29%, which is the highest among the shapes. It is noted that the conversion efficiency of the DSSCs with long gold nanorods added is higher than that of the cells with spherical gold nanoparticles. It may be because long gold nanorods have stronger surface plasma resonance effect on the TiO_2_ photoelectrodes than the spherical gold nanoparticles.

**Table 1:** The parameters of current–voltage characteristics for DSSCs without and with different shapes of gold nanoparticles

**Type**	***J***_**m**_	***V***_**m**_	***J***_**SC**_	***V***_**OC**_	**F.F.**	***η***
	**(mA/cm**^**2**^**)**	**(V)**	**(mA/cm**^**2**^**)**	**(V)**	**(%)**	**(%)**
Without	14.12	0.44	16.72	0.63	58.90	6.21
Nanosphere	15.41	0.44	18.20	0.64	58.37	6.77
Nanorod (AR 2.5)	15.72	0.45	18.24	0.65	59.99	7.08
Nanorod (AR 4.0)	16.19	0.45	18.30	0.65	61.23	7.29

**Figure 7:**
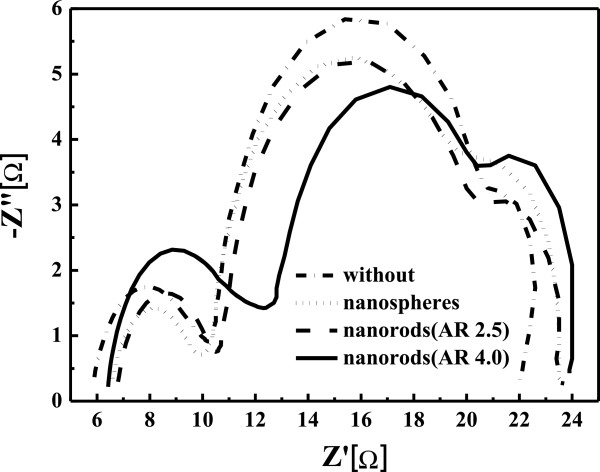
The spectra of EIS for the dye-sensitized solar cells without and with gold nanoparticles added.

**Figure 8:**
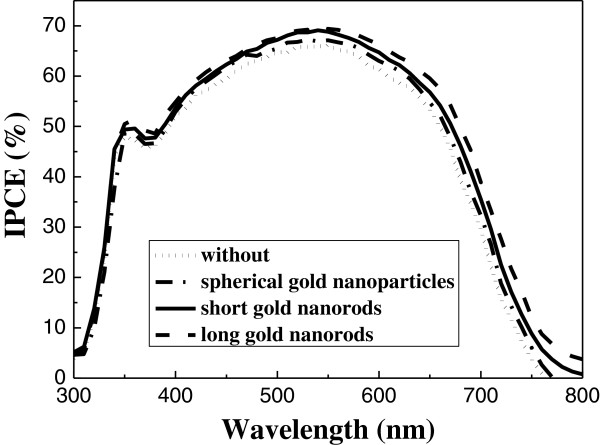
The IPCE spectra of DSSCs without and with gold nanoparticles added.

Figure 7 shows the spectra of EIS for the dye-sensitized solar cells without and with gold nanoparticles added. The simulation of the equivalent circuit is discussed in to the previous reports [18-20]. The parameter *R*_k_, which is the charge transfer resistance related to the recombination of electrons, is also listed in Table 2. The value of *R*_k_ decreases from 10.25 to 8.16 Ω when the long gold nanorods are added. It indicates that the effect of the long gold nanorods added in TiO_2_ film can improve the transport properties of TiO_2_ photoelectrodes, resulting in the increase of conversion efficiency of DSSCs. From the results of the current–voltage characterization and EIS, the effect of the gold nanoparticles on the TiO_2_ photoelectrodes can increase the conductivity and reduce the recombination of charges in the photoelectrodes, resulting in the increase of the conversion efficiency of the DSSCs. Furthermore, the long gold nanorods have stronger surface plasma resonance intensity than the spherical gold nanoparticles at long wavelength. This may be the reason why the conversion efficiency of the dye-sensitized solar cells with long gold nanorods is higher than those of the cells with spherical gold nanoparticles and short gold nanorods. Figure 8 shows the IPCE spectra of the DSSCs without and with gold nanoparticles added. The results of IPCE analysis indicate the number of incident photons inside the cells and their contribution to the efficiency. It is noted that all the IPCE spectra are similar in shape, and the IPCE value of the long gold nanorods is higher than those of the spherical gold nanoparticles and short gold nanorods in all wavelengths. It also provides an evidence that the conversion efficiency of DSSCs with long gold nanorods is higher than those of the cells with spherical gold nanoparticles and short gold nanorods.

**Table 2:** Characteristic parameters of the DSSCs without and with gold nanoparticles

**Type**	***κ***_**eff**_	***τ***_**eff**_	***R***_**s**_	***R***_**pt**_	***R***_**k**_
	**(S**^**-1**^**)**	**(S)**	**(Ω)**	**(Ω)**	**(Ω)**
Without	5.901	0.169	5.843	4.317	10.25
Nanosphere	5.258	0.190	6.602	3.325	9.80
Nanorod (AR 2.5)	5.1944	0.193	6.805	3.674	9.52
Nanorod (AR 4.0)	4.804	0.208	6.425	5.864	8.16

## Conclusions

In this study, we prepared different shapes of gold nanoparticles by the seed-mediated growth method to apply on the photoelectrodes of dye-sensitized solar cells. The diameter of the spherical gold nanoparticles is 45 nm, the length and width of the short gold nanorods are 55 and 22 nm, respectively, and the length and width of the long gold nanorods are 55 and 14 nm, respectively. The absorption spectrum of the TiO_2_ film with gold nanoparticles added is better than that of the film without gold nanoparticles, and the film with gold nanorods has stronger SPR intensity than that with spherical gold nanoparticles at long wavelength. This SPR effect results in higher conversion efficiency of the dye-sensitized solar cells with long gold nanorods those with spherical gold nanoparticles and short gold nanorods.

## Abbreviations

AA: Amino acid; AR: Aspect ratio; DI: Deionized; DSSC: Dye-sensitized solar cells; EIS: Electrochemical impedance spectroscopy; FE-SEM: Field emission scanning electron microscopy; IPCE: Incident photon conversion efficiency; UV-vis: Ultraviolet-visible absorption spectra.

## Competing interests

The authors declare that they have no competing interests.

## Authors’ contributions

THM and JKT wrote this manuscript. SMC, YCL, and TYC carried out the preparation of the samples. TCW, LWJ, and WW carried out the current–voltage measurements. WRC, ITT and CJH carried out the EIS and IPCE measurements. All authors read and approved the final manuscript.
